# Plasma Metabolomics Reveals Metabolic Profiling For Diabetic Retinopathy and Disease Progression

**DOI:** 10.3389/fendo.2021.757088

**Published:** 2021-10-29

**Authors:** Yu Sun, Huiling Zou, Xingjia Li, Shuhang Xu, Chao Liu

**Affiliations:** ^1^ Department of Endocrinology, Affiliated Hospital of Integrated Traditional Chinese and Western Medicine, Nanjing University of Traditional Chinese Medicine, Nanjing, China; ^2^ Department of Endocrinology and Metabolism, The Affiliated Suqian Hospital of Xuzhou Medical University, Suqian, China; ^3^ Treatment of Yingbing of State Administration of Traditional Chinese Medicine, Jiangsu Provincial Academy of Traditional Chinese Medicine, Nanjing, China

**Keywords:** diabetic retinopathy, plasma metabolomics, biomarkers, diabetes mellitus, machine learning

## Abstract

**Backgrounds:**

Diabetic retinopathy (DR), the main retinal vascular complication of DM, is the leading cause of visual impairment and blindness among working-age people worldwide. The aim of this study was to investigate the difference of plasma metabolic profiles in patients with DR to better understand the mechanism of this disease and disease progression.

**Methods:**

We used ultrahigh-performance liquid Q-Exactive mass spectrometry and multivariate statistical analyses to conduct a comprehensive analysis of plasma metabolites in a population with DR and proliferative DR (PDR). A risk score based on the level of the selected metabolite was established and evaluated using the least absolute shrinkage and selection operator regularization logistic regression (LASSO-LR) based machine learning model.

**Results:**

22 differentially expressed metabolites which belonged to different metabolic pathway were identified and confirmed to be associated with the occurrence of DR. A risk score based on the level of the selected metabolite pseudouridine was established and evaluated to strongly associated with the occurrence of DR. Four circulating plasma metabolites (pseudouridine, glutamate, leucylleucine and N-acetyltryptophan) were identified to be differentially expressed between patients with PDR and other patients, and a risk score formula based on these plasma metabolites was developed and assessed to be significantly related to PDR.

**Conclusions:**

Our work highlights the possible use of the risk score assessment based on the plasma metabolites not only reveal in the early diagnosis of DR and PDR but also assist in enhancing current therapeutic strategies in the clinic.

## Introduction

In both developing and developed countries, the prevalence of diabetes mellitus (DM) is rising. By 2045, it is estimated that 629 million people worldwide will have DM (1). The proportion was reported to be less than 1% in the 1980s in China, while a series of large-scale and well-conducted population surveys have shown that the prevalence has risen sharply to 9-12% in the past few years, with more than one million persons affected (2, 3). Diabetic retinopathy (DR), the main retinal vascular complication of DM, is the leading cause of visual impairment and blindness among working-age people worldwide (4). In addition, the existence of DR also suggests an increased risk of life-threatening systemic vascular complications (5, 6). By 2010, worldwide, DM-related eye disease contributed to the fifth most common cause of moderate-to-severe vision loss and blindness, accounting for nearly four million cases of visual impairment and more than eight hundred thousand cases of blindness (7).

DR is a progressive and devastating disease, and much of the blindness associated with DR can be prevented with early diagnosis and therapy. DR is classified according to its severity as nonproliferative DR (NPDR) in the early stages and proliferative DR (PDR) in the later stages; while PDR is often associated with visual impairment, NPDR is often asymptomatic (8). Therefore, profiling and early detection of DR are specifically vital in preventing NPDR from progressing to PDR. One challenge associated with the use of common retinal imaging methods widely utilized to screen and diagnose DR is training primary healthcare workers to assess these retinal images (9). However, an exciting area of research is the diagnosis and assessment of the occurrence, development and prognosis of disease based on liquid biopsy and the identification of easily accessible biomarkers; notably, plasma/serum multiomics can disclose systemic changes associated with biological dysfunction (10, 11). Thus, the development of DR diagnosis and PDR monitoring biomarkers must be advanced from a modern perspective so that treatment efforts for DR can be enhanced in the clinic.

Metabolomics research has been applied to qualitatively and quantitatively analyze all low-molecular-weight metabolites in a sample, identify metabolites with significant differences and important biological significance between different groups, and further clarify the metabolic processes and pathophysiological changes in an organism during the disease process (12, 13). This type of research moves from the genome to providing a complete illustration of the phenotype (14). Metabolomics is a powerful technology that can be leveraged to study biomarkers of various diseases, including DM. Several recent studies have revealed that the development of DM is closely related to amino acid metabolism, including that of branched chain amino acids, aromatic amino acids, tyrosine and other aromatic amino-containing acids, glycine, glutamine and glutamic acid (15). Despite the biological function of DM becoming increasingly apparent, the role of metabolites in regulating microvascular complications of DM such as DR remains under investigation.

In our work, we investigated plasma metabolomic biomarker profiling of DR patients and disease progression. We used ultrahigh-performance liquid Q-Exactive mass spectrometry (UPLC-QE-MS) and multivariate statistical analyses to conduct a comprehensive analysis of plasma metabolites in a population with DR and PDR. A risk score based on the level of the selected metabolite pseudouridine was established and evaluated using the least absolute shrinkage and selection operator regularization logistic regression (LASSO-LR) based machine learning method, and this score was strongly associated with the occurrence of DR. Subsequently, four circulating plasma metabolites (pseudouridine, glutamate, leucylleucine and N-acetyltryptophan) were identified to be differentially expressed between patients with PDR and other patients, and a risk score formula based on these plasma metabolites was developed and assessed to be significantly related to the severity of DR.

## Methods

### Chemicals and Reagents

HPLC-grade methanol was supplied by Tedia Company, Inc. (Fairfield, OH, USA). Formic acid (FA) was provided by Sigma-Aldrich Co., Ltd (St. Louis, MO, USA).

### Plasma Sample Collection

This study was managed at the The Affiliated Suqian Hospital of Xuzhou Medical University between August 2019 and January 2021, and the ethics committee of The Affiliated Suqian Hospital of Xuzhou Medical University (2019–102–07) approved the study, which was conducted according to the ethical standards for human experimentation and the World Medical Association (WMA) Declaration of Helsinki. The cases were T2DM patients with DR, and the controls were T2DM patients without DR. T2DM was diagnosed according to standard criteria recommended by WHO since 1999. All participants received detailed ophthalmic examinations and were separately assessed based on digital retinal photographs, while different stages of DR was diagnosed with fundus fluorescence angiography method (16). The inclusion criteria were as follows (1): T2DM (2); ≥18 years old (3); following the same therapy programs consists of basal insulin (Insulin Degludec) and metformin. Participants with following situation would be excluded (1): any other eye diseases or history of eye surgery (2); acute or chronic inflammatory disease, cardiovascular diseases, malignancy, liver or renal dysfunction and any other severe chronic systemic disease (3); poor quality of fundus photographs, which were not clear for DR diagnosis. According to inclusion criteria and exclusion criteria, 42 patients with clinical and histopathology-confirmed DR, including 21 PDR and 21 NPDR patients, and 32 age- and sex-matched T2DM patients without DR, were recruited between August 2019 and January 2021 at the Hospital of The Affiliated Suqian Hospital of Xuzhou Medical University. According to diabetic nephropathy diagnostic criteria (17), 6.25% (2/32) in T2DM group, 19.0 (4/21) in DR group and 61.9% (13/21) in PDR group were assessed to diabetic nephropathy.

### Sample Preparations for Metabolomics

Peripheral venous blood samples including 42 DR patients and 32 T2DM patients without DR, were drawn from the elbow vein in the fasting state in the morning and stored in ethylenediaminetetraacetic acid vacuum tubes (BD Vacutainer, Franklin Lakes, NJ, USA) and then centrifuged at 1300 g for 10 min at 4°C. Plasma was immediately separated and stored at -80°C. Sample preparation for nontargeted metabolomics was performed according to the manufacturer’s instructions. One hundred microliters of each sample were slowly lysed at 4°C, 400 µL of precooled methanol was added, vortexed for 60 s, and incubated at -80°C for 8 hours, and the protein was precipitated by centrifugation at 16,000 g for 10 min at 4°C. The supernatant was used for UHPLC analysis.

### Metabolite Profile Analysis and Metabolite Identification

The samples were separated by UHPLC and then analyzed by a Thermo QE HF-X mass spectrometer carried out by Clinical Mass Company (Nanjing, China). Electrospray ionization (ESI) positive and negative ion modes were applied. The ESI source conditions after C18 chromatographic separation were as follows: sheath gas flow rate: 50; Aux gas flow rate: 13; sweep gas flow rate: 0; capillary temperature: 300°C; spray voltage: ± 3.5 kV; scan m/z range: 67-1000 Da; and product ion scan m/z range: 67-1000 Da. Secondary mass spectra were obtained using information-dependent acquisition (IDA) and high sensitivity mode, with an N collision energy of 15, 30, 45 eV. Aliquots of samples were mixed for the preparation of QC samples. QC samples were inserted in the sample cohort throughout the analysis to monitor and evaluate system stability. The MSdial program was used for peak extraction of the data, and the SIMCA program was used for principal component analysis (PCA) and orthogonal partial least-squares discriminant analysis (OPLS-DA). Then, metabolite structure identification was performed by exact mass number matching and secondary spectrum matching by searching public databases.

### Correlation-Based Metabolic Network Analysis and Metabolic Pathway Analysis

The MS signal intensities confirmed that significantly changed metabolites were converted by log transformation and autoscaling and applied to calculate Pearson’s correlation coefficient, followed by correlation-based metabolic networking analysis using Cytoscape 3.7. Variable metabolites were imported and analyzed using MetaboAnalyst software (http://www.metaboanalyst.ca) to perform pathway analysis to display the role of disturbed metabolic pathways.

### Biochemical Measurements

All patients’ medical histories were acquired, and age, sex, body mass index (BMI), and duration were obtained after a physical examination. Patients underwent blood and urine laboratory tests that included fasting plasma glucose (FPG), glycated hemoglobin |glycosylated hemoglobin (HbA1c), urine Albumin to creatinine (UACR), triglycerides, high-density lipoprotein cholesterol (HDL-c), low-density lipoprotein cholesterol (LDL-c) and total cholesterol (TC).

### Statistical Analysis

Data are presented as the mean ± SD. Continuous data were analyzed with Student’s t-test or the Mann-Whitney U test using SPSS 22.0 software (SPSS, Chicago, IL, USA). As the association of HbA1c level with severity of retinopathy has been investigated and assessed, Pearson correlation and partial correlation were used to analyze the relationships between plasma HbA1c level and risk score (GraphPad Prism). LASSO-LR based machine learning model was then performed to derive an DR or PDR diagnosis risk score. Receiver operating characteristic (ROC) analysis was utilized to estimate the sensitivity and specificity by the standard method. The general acceptance level of significance was P < 0.05.

## Results

### Clinical Features of Subjects

In the present work, we explored the association between the plasma metabolite fingerprint and proliferative retinopathy in DM patients. Detailed demographic characteristics of the enrolled participants are shown in **Table 1**, FPG, HbA1c and UACR levels were markedly higher in DR patients (P < 0.001). In addition, no significant differences were found for BMI, or levels of triglycerides, HDL-c, LDL-c and TC between the DR and control groups (P > 0.05).

**Table 1:** Detailed demographics of the enrolled patient.

Detailed demographics of the enrolled patients		
	DR	NDR
n	42	32
Gender (male/female)	18/24	15/17
Age (years)	52 (45–62)	50 (45–61)
Dibabets duration (years)	13 (11.4-19)	12.5 (10.5-18.5)
BMI (kg/m2)	26.8 (23.8-29.4)	25.4 (22.3-28.9)
triglycerides (mmol/L)	1.3 (0.78-1.9)	1.7 (0.86-2.3)
HDL-c (mmol/L)	0.89 (0.59-1.23)	0.92 (0.63-1.29)
LDL-c (mmol/L)	2.96 (2.03-3.61)	2.78 (2.13-3.53)
TC (mmol/L)	4.86 (3.62-5.52)	4.72 (3.30-5.38)
FPG (mmol/L)	10.05 (8.97-11.31)	8.11 (6.71-8.93)
UACR (mg/g)	37.4 (6–213)	17.3 (4.1-45.2)
HbA1c (1%)	9.47 (8.78-10.69)	8.03 (7.58-8.63)

DR, Diabetic retinopathy; BMI, body mass index; FPG, fasting plasma glucose; HbA1c, glycated hemoglobin glycosylated hemoglobin; HDL-c, high-density lipoprotein cholesterol; LDL-c, low-density lipoprotein cholesterol; TC, total cholesterol; UACR, urine albumin to creatinine.

### Metabolomics Workflow

**Figure 1:**
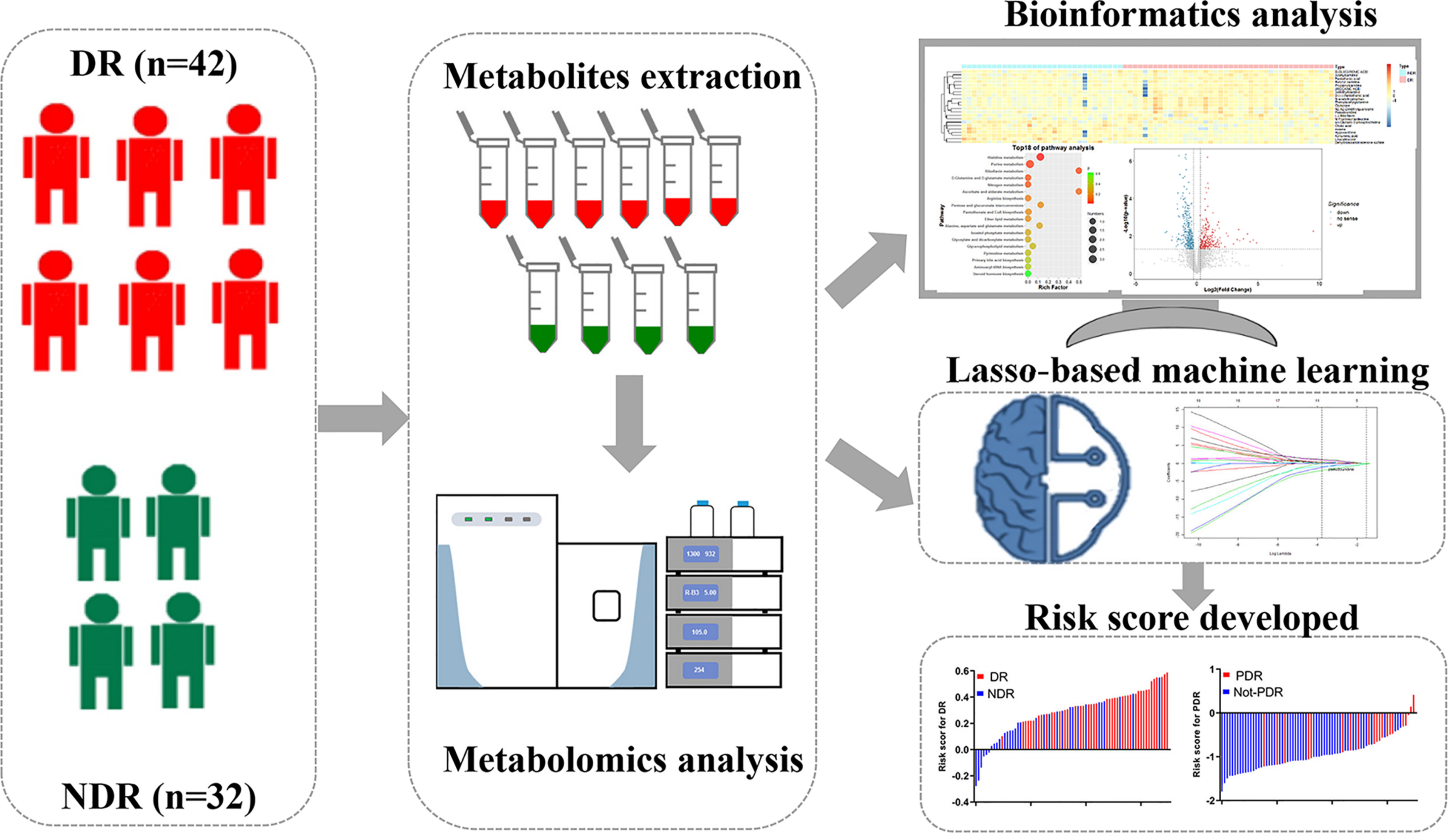
Workflow of metabolomics for metabolomic profiling and data interpretation of plasma samples from DR and NDR.

The study workflow is shown in **Figure 1**. Plasma samples were collected from subjects and analyzed with the UHPLC-QE MS platform with both the electrospray ionization positive (ESI+) and negative (ESI-) modes. Raw data were normalized using Pareto scaling for subsequent data analysis after extraction of the background and alignment of the metabolic peaks. Different metabolic features and metabolites were extracted by combining the criteria of fold change (FC) >1.2 and *P*<0.05 and visualized with volcano plots and heat maps. Thirty significantly different metabolites were screened by the threshold of variable important in projection (VIP) value >1 and *P* value <0.05, of which correlation analysis and pathway analysis were performed. The LASSO-LR was utilized to select diagnostic markers to predict DR and PDR among the subjects *via* penalized maximum likelihood. Evaluation of the risk score formula was performed using ROC analysis, and the relationship between the risk score and HbA1c level was investigated.

### Metabolic Profiling of DR

**Figure 2:**
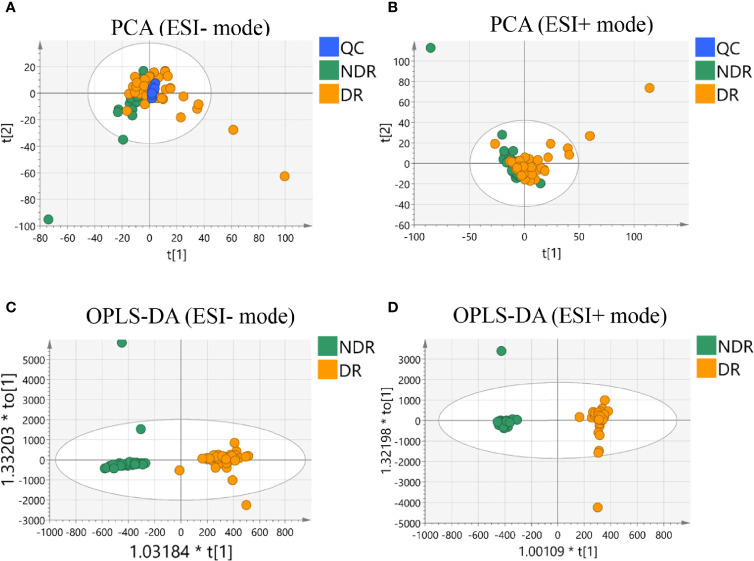
Multivariate statistical analysis results. PCA score plot of the analysis in ESI (–) mode **(A)** and ESI (+) mode **(B)**. OPLS-DA score plot of the analysis in ESI (–) mode **(C)** and ESI (+) mode **(D)**.

We first compared the metabolic signatures between DR and control T2DM patients in both ESI+ and ESI− modes of untargeted metabolomics. In total, more than 50,000 metabolic features were consistently found in all plasma samples from the discovery cohort, including 25742 features in ESI+ mode and 31374 features in ESI- mode. QC samples were tightly clustered in principal component analysis (PCA), validating the stability and reproducibility of the instrumental analysis (**Figures 2A, B**). The OPLS-DA score plot displays a clear demarcation between the DR group and the control group in ESI+ mode with R^2^Y = 0.939 and in ESI- mode with R^2^Y = 0.991, suggesting significant changes in plasma metabolites in the DR group (**Figures 2C, D**).

### Identification of Differential Metabolites

**Figure 3:**
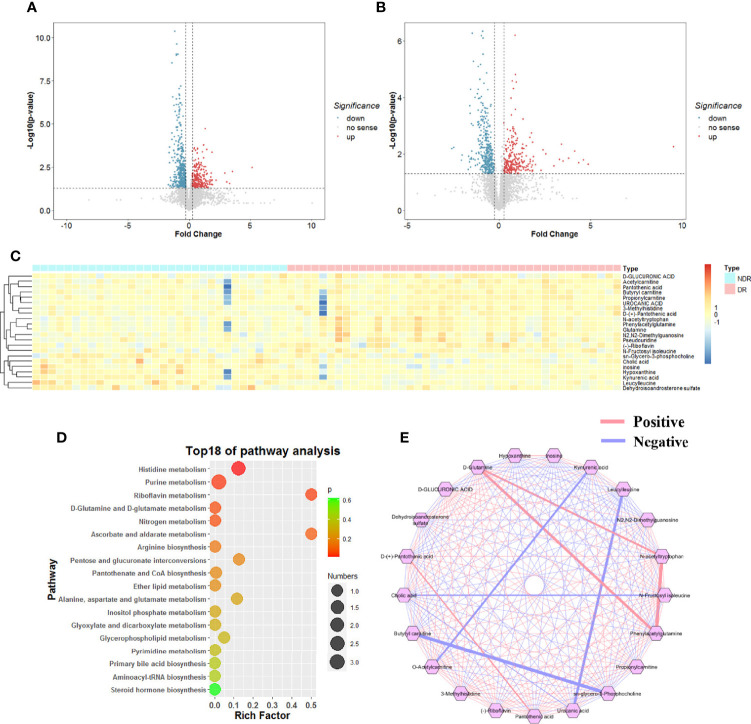
Representative Volcano plot (fold change >1.2 and p-value < 0.05) in ESI (+) mode **(A)** and ESI (+) mode **(B)** metabolomics data. **(C)** Representative heatmap of significant different metabolites (fold change >1.2, VIP>1 and p-value < 0.05). **(D)** Correlation-based metabolic network analysis. **(E)** Metabolic pathway analysis.

To reveal the plasma metabolic characteristics in DR patients and identify and confirm high-confidence metabolites that contribute to DR, we distinguished the differences by ESI+ and ESI- based on the criteria of FC>1.2 and P<0.05, respectively. In addition, a VIP value greater than 1.0, which was calculated by OPLS-DA scoring, was selected as a significantly different metabolic feature for analysis. Thus, metabolic characteristics with significant differences were extracted and visualized by volcano plots (**Figures 3A, B**). According to a public metabolite library, 22 metabolites were identified and confirmed after inputting the refined significant metabolic features, containing 13 and 5 metabolites from the ESI+ and ESI- models, respectively, and 4 metabolites with dual mode. They were classified into 13 subcategories according to the chemical taxonomy in the Human Metabolome Database (HMDB) with the largest proportion of the significantly different metabolites which was classified as amino acid (7/22) (**Table 2**). Hierarchical clustering analysis also revealed differentially expressed metabolites between DR and NDR (**Figure 3C**).

**Table 2:** Differential metabolites identified from metabolomics profiling.

Metabolite name	Mode	VIP	FC	P Value	Subclass
Pantothenic acid	POS	1.53	1.553	0.0057	vitamin
(–)-Riboflavin	POS	2.05	2.819	0.0076	vitamin
D-(+)-Pantothenic acid	NEG	1.16	1.357	0.0310	vitamin
Pseudouridine	NEG	1.63	1.720	0.0047	uridine
D-GLUCURONIC ACID	NEG	1.30	1.316	0.0455	sugars
Dehydroisoandrosterone sulfate	NEG	1.19	0.598	0.0456	steroids
Hypoxanthine	NEG/POS	2.60	0.360	0.0079	purine derivatives
N2,N2-Dimethylguanosine	POS	1.11	1.439	0.0331	nucleoside
sn-Glycero-3-phosphocholine	POS	1.01	0.705	0.0301	lipid
Propionylcarnitine	POS	1.05	1.276	0.0267	lipid
Acetylcarnitine	POS	1.38	1.584	0.0088	enzyme
Inosine	NEG/POS	2.18	0.315	0.0363	creatinine
Cholic acid	NEG/POS	2.53	0.244	0.0172	cholic acid
Butyryl carnitine	POS	1.48	1.561	0.0024	carnitine
UROCANIC ACID	POS	1.33	1.373	0.0002	azole
N-Fructosyl isoleucine	POS	1.09	1.496	0.0474	amino acid
N-acetyltryptophan	POS	1.95	3.762	0.0341	amino acid
Leucylleucine	POS	3.24	0.329	0.0002	amino acid
Kynurenic acid	POS	1.90	0.541	0.0000	amino acid
3-Methylhistidine	POS	1.86	2.264	0.0010	amino acid
Phenylacetylglutamine	NEG/POS	2.10	3.262	0.0188	amino acid
Glutamine	NEG	1.98	2.560	0.0196	amino acid

POS, Positive; NEG, Negative; FC, Fold change; VIP, Variable important in projection.

### Metabolite Correlation Analysis and Pathway Enrichment Analysis

To further investigate the interrelationships between the significantly different metabolites, we utilized the Metscape plugin (http://metscape.ncibi.org/) in Cytoscape (https://cytoscape.org/), a tool available for interactive exploration and visualization of metabolic networks in metabolite changes, and constructed the metabolic network (**Figure 3D**). We identified 45 pairs of correlations with correlation coefficients ≥0.4 or ≤-0.4 among the 22 significantly different metabolites. KEGG pathway enrichment analysis was performed for 22 dysregulated metabolites involving 18 metabolic pathways. Based on the enrichment factor and P-value, histidine metabolism, purine metabolism, riboflavin metabolism, d-glutamine metabolism and nitrogen metabolism were the five most significantly enriched metabolic pathways (**Figure 3E**).

### Development and Evaluation of a Diagnostic Panel for DR

**Figure 4:**
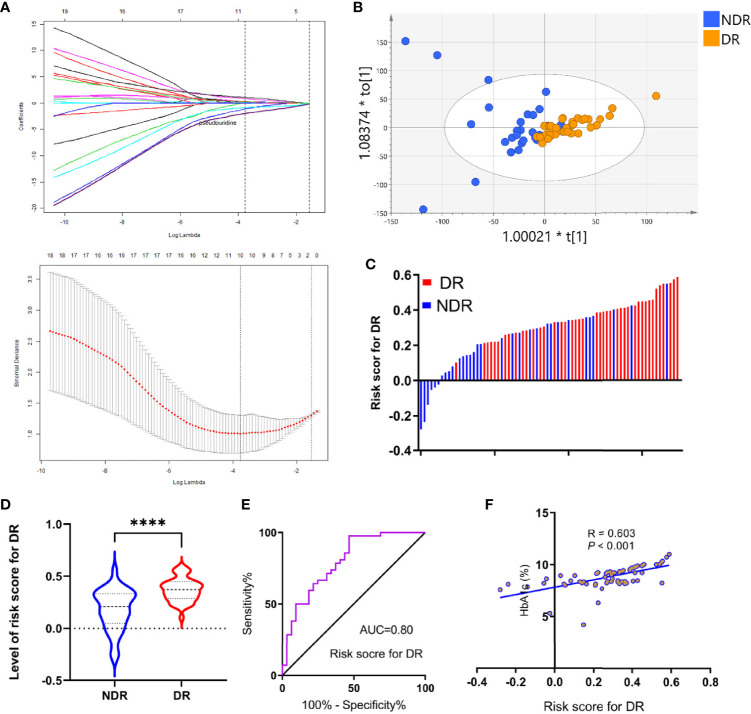
Development of risk score for DR using the least absolute shrinkage and selection operator regularization (LASSO-LR) model. **(A)** Dotted vertical lines were drawn at the optimal values with Lambda (log), by using the minimum criteria and the 1 standard error of the minimum criteria (the 1-SE criteria). **(B)** OPLS-DA score plot of the analysis using selected metabolite. **(C)** Distribution of the risk score in the group. **(D)** Statistical analysis for distribution of risk score between DR and NDR (^****^p<0.0001). **(E)** ROC curves were created to evaluate the power of risk score. **(F)** A linear correlation analysis between risk score and HbA1c levels.

To further elucidate the metabolite signature for DR, a LASSO-LR model was utilized to select diagnostic metabolites to predict DR among the subjects *via* penalized maximum likelihood, which gives the most normalized model for the application of one or ten markers (**Figure 3A**). The normalization path was calculated for the LASSO-LR model at a grid of values for the normalization parameter lambda, which identified one (pseudouridine) or ten differentially expressed metabolites (**Figure 4A**). The OPLS-DA score plot displays a clear demarcation between the DR group and the control group at the level of pseudouridine with R2Y = 0.867 (**Figure 4B**). According to the levels of the selected metabolites, the following formula was derived to calculate the DR risk score for each patient: risk score (DR) = -0.23× Ln (pseudouridine) + 1.88. Based on the study of the relationship between the risk score distribution and DR status, the results showed that the rate of DR in the low-risk score party was primarily lower than that in the high-risk score party (**Figure 4C**). In addition, the risk scores for the DR group were predominantly higher than those for the NDR group (**Figure 4D**). The sensitivity and specificity of the risk score for DR were 97.6% and 53.1%, respectively, with an AUC of 0.80 (95% CI = 0.70 to 0.90) (**Figure 4E**). Then, it was also shown that the risk score was positively correlated with the level of HbA1c according to a linear correlation analysis (R = 0.603, P < 0.001) (**Figure 4F**).

### Plasma Metabolomics Approach to Monitor the Progression of DR

**Figure 5:**
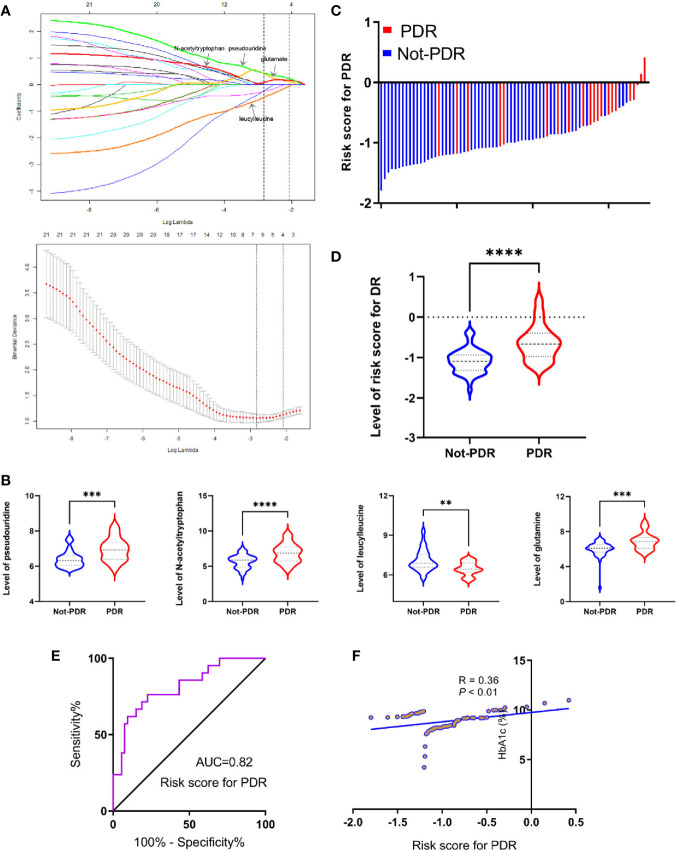
Development of risk score for PDR using the least absolute shrinkage and selection operator regularization (LASSO-LR) model. **(A)** Dotted vertical lines were drawn at the optimal values with Lambda (log), by using the minimum criteria and the 1 standard error of the minimum criteria (the 1-SE criteria). **(B)** Statistical analysis of pseudouridine, glutamate, leucylleucine and N-acetyltryptophan between PDR and not-PDR group (^**^p < 0.01; ^***^p < 0.001; ^****^p < 0.0001). **(C)** Distribution of the risk score in the group. **(D)** Statistical analysis for distribution of risk score between PDR and not-PDR group (^****^p < 0.0001). **(E)** ROC curves were created to evaluate the power of risk score. **(F)** A linear correlation analysis between risk score and HbA1c levels.

According to the International Clinical DR and Diabetic Macular Edema Disease Severity Scale, 21 PDR patients and 53 non-PDR patients, including 32 control cases and 21 NPDR patients, were distinguished. Then, the same procedure of LASSO-LR analysis was performed to select metabolites to monitor PDR. The normalization path was calculated for the LASSO-LR model at a grid of values for the normalization parameter lambda, which identified four (pseudouridine, glutamate, leucylleucine and N-acetyltryptophan) differentially expressed metabolites (**Figure 5A**). As shown in **Figure 5B**, the plasma concentrations of pseudouridine, N-acetyltryptophan and glutamate were predominantly upregulated, whereas that of leucylleucine was found to be downregulated in PDR patients. According to the levels of the four selected metabolites, the following formula was derived to calculate the PDR risk score for each patient: risk score = 0.23 × Ln(pseudouridine) + 0.16 × Ln(N-acetyltryptophan) - 0.065 × Ln(leucylleucine) + 0.11 × Ln(glutamate) -3.63. Based on the relationship between the risk score distribution and DR status, the results showed that the rate of PDR cases in the low-risk score party was primarily lower than that in the high-risk score party (**Figure 5C**). Statistical analysis showed that the risk scores of the PDR group were significantly higher than those of the non-PDR group (**Figure 5D**). The sensitivity and specificity of the risk score for DR were 76.2% and 77.4%, respectively, with an AUC of 0.82 (95% CI = 0.71 to 0.90) (**Figure 5E**). Ultimately, it was also shown that the risk score was positively correlated with the level of HbA1c according to a linear correlation analysis (R = 0.36, P < 0.01) (**Figure 5F**).

## Discussion

DR, the main retinal vascular complication of DM, is the leading cause of visual impairment and blindness among working-age people worldwide. Therefore, early and accurate identification of DR and disease progression among T2DM patients is essential for clinicians when evaluating the disease status of patients and formulating suitable therapy efforts such as anti-vascular endothelial growth-factor agents, intraocular injection of steroids or timely laser therapy for preservation of sight in DR patients. In our work, we demonstrated that the levels of circulating plasma metabolites were significantly differentially expressed between the DR and NDR groups. A risk score based on the level of pseudouridine was established and evaluated using the LASSO-LR model, which was strongly associated with the occurrence of DR. Subsequently, four circulating plasma metabolites, including pseudouridine, glutamate, leucylleucine and N-acetyltryptophan, were identified to be differentially expressed between PDR and not-PDR, and a risk score formula based on these four plasma metabolites was developed in the same way and assessed to be significantly related to the severity of DR. Our work highlights the possible use of plasma metabolites in the early diagnosis of DR and PDR in the clinic.

A panel of differentially expressed plasma metabolites was first identified after comparing DR and NDR subjects and was found to be significantly enriched in histidine metabolism, purine metabolism, riboflavin metabolism, d-glutamine/d-glutamate metabolism and others. Histidine, an essential amino acid (EAA) in mammals, is derived from growth and amino acid composition in tissues. An increasing number of studies have revealed the effect of histidine catabolism on carnosine synthesis, which contributes to strong antioxidant effects (18) and the efficiency of chemotherapy agents (19), preventing cataracts (20). The major pathway of histidine catabolism could proceed in four steps to yield glutamate, with many functions, including the development of DM (21). Our data are in accordance with previous studies showing that purine metabolism plays a role in the development of DR (22). Purines, the basic composition of nucleotides in the process of cell proliferation, are associated with various molecular functions, such as cell cycle regulation, signal transduction and immune function. Uric acid, the final product of purine metabolism, is an independent predictor of cardiac allograft vasculopathy after heart transplantation (23). Additionally, uric acid has been reported to be a risk factor related to extremity vasculopathy in T2DM (24). Riboflavin, a water-soluble vitamin, is an essential nutrient in higher organisms and is involved in oxidation-reduction reactions in many metabolic pathways and in energy production in the respiratory chain that occurs in the mitochondria. Impairment of flavin homeostasis may lead to multisystem dysfunction, including neuromuscular disorders and cardiovascular disease (25). Moreover, diabetic patients have been reported to suffer from riboflavin deficiency, and flavin imbalance plays a vital role in the appearance of DR (26). The above evidence indicates that metabolic pathways may contribute to the process of DR.

Our results also showed that the pseudouridine gradient increased in DR and PDR subjects. From screening and fitting results based on LASSO-LR analysis, variable selection and regularization were performed when fitting a generalized linear curve. The variable profiling here mentions not setting all the variables into the model for fitting but to selectively set the variables into the model to obtain better performance parameters to avoid overfitting (27). Through LASSO-LR based machine learning methods, the optimal number of metabolites can be screened from a large number of variables, and equations based on these metabolites can be established. To our knowledge, there has been no metabolomics research on the impact of pseudouridine in patients who have undergone DR and disease progression. It has generally been acknowledged that pseudouridine, a fundamental metabolite, is a c-glycosyl pyrimidine that consists of uracil having a beta-D-ribofuranosyl residue attached at position 5. Pseudouridine is also associated with RNA modification (28), owing to the relative abundance and inertness of the isomer compared with other mNS in cells, and comprises approximately 5% of all cellular RNA nucleotides (29). The unique structural properties of pseudouridine contribute to the folding of tRNAs and rRNA, and recent research suggests that pseudouridylation influences the coding potential of mRNA. Recently, pseudouridine has been identified and observed in the plasma, urine or tissue of cancer patients with multiple malignancies, including prostate cancer (30), hepatocellular carcinoma lymphoma (31), colorectal cancer (32) and chronic kidney disease (33). Pseudouridine has also been shown to be a novel diagnostic metabolic marker of heart failure, which is similar to the observation of the Alexander D team, who found that pseudouridine was elevated in dilated cardiomyopathy patients (34). Besides, the relationship of pseudouridine and the risk of diabetes had been disclosed. Pseudouridine inhibits glucose utilization at the postreceptor level through lowering the intracellular Ca concentration to affect the progression of T2DM (35), and plasma pseudouridine predict both the risk and prevalence (36) and insulin resistance of T2DM (37). Moreover, plasma pseudouridine has been shown to be correlated with declining renal function and albuminuria in diabetic kidney disease (38, 39), suggesting a close relationship between the level of plasma pseudouridine and diabetic microangiopathy.

Glutamate, another metabolite that was shown to be associated with DR and PDR in our work, is the most abundant and versatile amino acid in the body. Under normal conditions, glutamate is the principal excitatory neurotransmitter in the brain and is involved in learning and memory (40). In addition, glutamate may play a role in acute brain damage after traumatic brain injury, cerebral ischemia and status epilepticus (41, 42), immune system (43), the endocrine system (44), kidney and coronary artery disease (45) and others. The roles of plasma glutamine acid in DR and disease progression have not yet been illustrated. However, some evidence has revealed their effect on the development of DM and DM-related complications. Glutamate is significantly associated with the risk of developing T2DM (45, 46). In addition, other studies have suggested that DM is accompanied by an accumulation of glutamate in the retina, which causes neurotoxicity and the development of DR (47, 48), while glutamine is regarded as the most individual metabolite for the presence of DR (49).

There were a few limitations in our study, one of which is its small sample size with only 78 plasma samples applicable for metabolomic analysis, which is unfavorable for investigating the robustness of the model. Therefore, the sensitivity and specificity of the diagnostic model should be assessed with an expanded number of patients as well as in a prospective cohort. In addition, the absolute concentration of candidate metabolites was not quantified and validated in our study, making them difficult to apply in the clinic.

In brief, liquid biopsy metabolomics could be applied to discriminate metabolic subphenotypes of DR and disease progression, with the identification and validation of specific circulating discriminant metabolites. Based on the aforementioned results, we were able to develop a risk score according to the level of metabolites for DR and PDR. Further investigations are required to quantitatively detect candidate metabolites in an expanded cohort. Nevertheless, our work demonstrated that this risk score based on molecular signatures should enable the monitoring of the appearance of disease and disease progression at an early stage.

## Data Availability Statement

The original contributions presented in the study are included in the article/supplementary material. Further inquiries can be directed to the corresponding authors.

## Ethics Statement

Written informed consent was obtained from the individual(s) for the publication of any potentially identifiable images or data included in this article.

## Author Contributions

CL and SX conceived of and designed the experiments. YS, HZ, and XL performed the experiments. YS and HZ analyzed the data and YS wrote the manuscript. All authors contributed to the article and approved the submitted version.

## Conflict of Interest

The authors declare that the research was conducted in the absence of any commercial or financial relationships that could be construed as a potential conflict of interest.

## Publisher’s Note

All claims expressed in this article are solely those of the authors and do not necessarily represent those of their affiliated organizations, or those of the publisher, the editors and the reviewers. Any product that may be evaluated in this article, or claim that may be made by its manufacturer, is not guaranteed or endorsed by the publisher.
